# Vitamin D and C-Reactive Protein: A Mendelian Randomization Study

**DOI:** 10.1371/journal.pone.0131740

**Published:** 2015-07-06

**Authors:** Marte C. Liefaard, Symen Ligthart, Anna Vitezova, Albert Hofman, André G. Uitterlinden, Jessica C. Kiefte-de Jong, Oscar H. Franco, M. Carola Zillikens, Abbas Dehghan

**Affiliations:** 1 Department of Epidemiology, Erasmus University Medical Center, Rotterdam, the Netherlands; 2 Netherlands Institute for Health Sciences, Rotterdam, the Netherlands; 3 Department of Internal Medicine, Erasmus University Medical Center, Rotterdam, the Netherlands; University of Leicester, UNITED KINGDOM

## Abstract

Vitamin D deficiency is widely prevalent and has been associated with many diseases. It has been suggested that vitamin D has effects on the immune system and inhibits inflammation. The aim of our study was to investigate whether vitamin D has an inhibitory effect on systemic inflammation by assessing the association between serum levels of vitamin D and C-reactive protein. We studied the association between serum 25-hydroxyvitamin D and C-reactive protein through linear regression in 9,649 participants of the Rotterdam Study, an observational, prospective population-based cohort study. We used genetic variants related to vitamin D and CRP to compute a genetic risk score and perform bi-directional Mendelian randomization analysis. In linear regression adjusted for age, sex, cohort and other confounders, natural log-transformed CRP decreased with 0.06 (95% CI: -0.08, -0.03) unit per standard deviation increase in 25-hydroxyvitamin D. Bi-directional Mendelian randomization analyses showed no association between the vitamin D genetic risk score and lnCRP (Beta per SD = -0.018; p = 0.082) or the CRP genetic risk score and 25-hydroxyvitamin D (Beta per SD = 0.001; p = 0.998). In conclusion, higher levels of Vitamin D are associated with lower levels of C-reactive protein. In this study we did not find evidence for this to be the result of a causal relationship.

## Introduction

Low vitamin D levels are present in up to 50% of the adult population in developed countries.[1] The most important causes for low vitamin D are lack of sun exposure, which leads to inadequate production of the precursor of vitamin D in the skin, and insufficient nutritional intake. The vitamin D receptor is present on immune cells, such as monocytes and T-helper cells. Therefore it is speculated that vitamin D could have effect on immune response and chronic inflammation.[2–4] Inflammation is known to be involved in several complex disorders, potentially through its influence on cell growth, tissue damage, pancreatic beta-cell failure and the development of atherosclerosis.[5] Previous studies investigating the association between vitamin D and inflammation have shown inconsistent results. [6–15] Some studies found inverse associations between serum vitamin D and inflammatory markers, yet due to the observational nature of these studies the question of causality remains unanswered.[8, 9]

**Fig 1:**
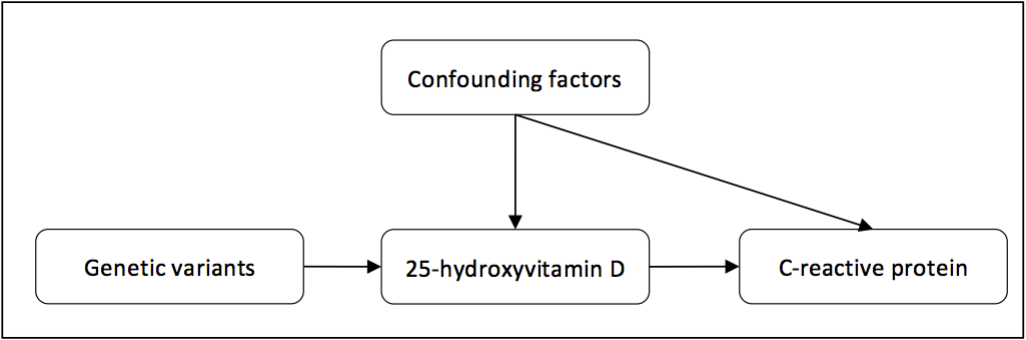
Concept of Mendelian randomization.

Conclusions about causality cannot be drawn merely based on the presence of an association in an observational design. A complementary alternative is to apply the Mendelian randomization approach, in which the relationship between a genetic determinant of a predictor variable and a specific outcome is studied (Fig 1).[16, 17] If there is indeed a causal effect of vitamin D on inflammation as measured with C-reactive protein (CRP), genetic determinants related to vitamin D should be associated with CRP levels In turn, if inflammation would lower vitamin D levels, genetic determinants of CRP would be expected to be associated with vitamin D levels. These associations are less prone to confounding, since the genetic variants are inherited randomly and do not associate with any other factors. Moreover, reverse causation is unlikely, due to the constant nature of genetic variants over their life course.[16, 17]

We investigated the association between serum 25-hydroxyvitamin D and CRP in the Rotterdam Study, a prospective population–based cohort. Furthermore, we evaluated a potential causal effect by using genetic variants in bi-directional Mendelian randomization analysis.

## Methods

### Study population

This study was conducted among participants of the first (RSI), second (RSII) and third (RSIII) cohort of the Rotterdam Study, a prospective population-based cohort study that has been ongoing since 1989 in the district of Ommoord in the city of Rotterdam, The Netherlands. The design of this study has been described previously. [18, 19] In brief, residents aged 55 and over living in the district of Ommoord in Rotterdam, the Netherlands, were invited to participate. Seventy-eight percent of the invitees agreed to participate and were included in the first study cohort (n = 7,983). In 1999 the study was extended with a second cohort, comprising 3,011 subjects that had reached the age of 55 years and over. Finally, a third cohort consisting of 3,932 subjects aged 45 and over was included in 2006, after which the study population totals 14,926 subjects.

The study was approved by the medical ethics committee at Erasmus University Rotterdam. All participants gave written informed consent.

### 25-hydroxyvitamin D

Plasma levels of 25-hydroxyvitamin D were measured in non-fasting samples of 1,428 subjects at the first visit of RSI (RSI-1) and 3,799 samples at the third visit of RSI (RSI-3), of which 1,323 were overlapping. Plasma 25-hydroxyvitamin D was measured in fasting samples of 2,464 and 3,420 subjects at the first visits of RSII (RSII-1) and RSIII (RSIII-1) respectively.

In RSI-1, 25-hydroxy vitamin D (25OHD) serum levels were measured using a radioimmunoassay (IDS Ltd, Boldon, UK, available at www.idsltd.com). This test detects levels within a range of 4 to 400 nmol/l, with a sensitivity of 3 nmol/l, a within-run precision <8% and a total precision <12%. Measurements in RSI-3, RSII-1 and RSIII-1 were done using an electrochemiluminescense-based assay (Elecsys Vitamin D Total, Roche Diagnostics, Mannheim, Germany). This test detects levels within a range of 7.50–175 nmol/l, with a sensitivity of 10 nmol/l, a within-run precision <6.5% and a total precision <11.5%.

### C-reactive protein

At RSI-1, plasma levels of CRP were measured in non-fasting samples of 6,569 subjects, and at RSI-3 in 3,986 subjects, of which 3,694 were overlapping. The samples were put on ice immediately and were processed within 30 minutes. Samples were kept frozen at -20°C until CRP was measured. High-sensitivity CRP was measured using a rate near-infrared particle immunoassay (IMMAGE Immunochemistry System, Beckman Coulter, Fullerton, CA). This system detects concentrations from 0.2 to 1,440 mg/l, with a within-run precision <5.0%, a total precision <7.5%, and a reliability coefficient of 0.995.

In RSII-1 and RSIII-1, plasma levels of CRP were measured in fasting samples of 2,512 and 3,440 subjects respectively. CRP was measured using a particle enhanced immunoturbidimetric assay (Roche Diagnostics, Mannheim, Germany), which detects concentrations from 0.3–350 mg/l, with a sensitivity of 0.6 mg/l.

### Genotyping

Genotyping was done using genomic DNA extracted from peripheral venous blood samples according to standard procedures. Genotyping was performed with the version 3 Illumina Infinium HumanHap 550K chip RSI and RSII and the Illumina Infinium HumanHap 610 Quad chip in RSIII. SNPs with allele frequency ≤1%, Hardy–Weinberg equilibrium P<10^−6^, or SNP call rate <98% were excluded. Imputation was performed with 1000 Genome phase I, version 3 as the reference panel using the maximum likelihood method implemented in MACH. [20, 21] We selected four vitamin D related SNPs based on a genome-wide association study (GWAS) on serum 25-hydroxyvitamin D. [22] For C-reactive protein, we selected 18 SNPs from the latest available GWAS on serum C-reactive protein. [23] The selected SNPs are depicted in Table 1.

**Table 1:** SNPs associated with 25-hydroxyvitamin D or C-reactive protein.

SNP	Associated with	Risk Allele^*^	Nearest Gene
rs12785878	25-hydroxyvitamin D	G	DHCR7
rs10741657	25-hydroxyvitamin D	G	CYP2R1
rs2282679	25-hydroxyvitamin D	G	GC
rs6013897	25-hydroxyvitamin D	A	CYP24A1
rs2794520	C-reactive protein	C	CRP
rs4420638	C-reactive protein	A	APOC1
rs1183910	C-reactive protein	G	HNF1A
rs4420065	C-reactive protein	C	LEPR
rs4129267	C-reactive protein	C	IL6R
rs1260326	C-reactive protein	T	GCKR
rs12239046	C-reactive protein	C	NLRP3
rs6734238	C-reactive protein	G	IL1F10
rs9987289	C-reactive protein	A	PPP1R3B
rs10745954	C-reactive protein	A	ASCL1
rs1800961	C-reactive protein	C	HNF4A
rs340029	C-reactive protein	T	RORA
rs10521222	C-reactive protein	C	SALL1
rs12037222	C-reactive protein	A	PABPC4
rs13233571	C-reactive protein	C	BCL7B
rs2847281	C-reactive protein	A	PTPN2
rs6901250	C-reactive protein	A	GPRC6A
rs4705952	C-reactive protein	G	IRF1

### Covariates

Body Mass Index (BMI) was calculated as weight in kilogram divided by the square height in meters. Height and body weight were measured while the participants wore indoor clothing and no shoes. Blood pressure was defined as the mean of two consecutive measurements, which were obtained by trained research assistants from the right brachial artery, with the patient in a sitting position.

Total cholesterol and high-density lipoprotein were measured with standard laboratory techniques, after which the TC/HDL ratio was calculated. Prevalent diabetes mellitus was defined as a fasting serum glucose ≥7.0 nmol/l, a non-fasting serum glucose ≥ 11.1 nmol/l and/or use of anti-diabetic medication. The abbreviated modification of diet in renal disease (MDRD) equation was used to estimate glomerular filtration rate.[24] Smoking habits were divided in three categories: former smoker, current smoker and never smoker. Information on current health status, medical history, medication use, alcohol use, smoking behavior and education was obtained by trained research assistants during home visits. Level of education was categorized according to the International Standard Classification of Education. [25] Bone mineral density measurement of the femoral neck was performed by dual energy X-ray absorptiometry (DXA) (Lunar DPX-L densitometer, Madison, WI, USA).[26] From these measurements, sex-specific T-scores were calculated using the NHANES reference population of Caucasian males and females aged 20 to 29 years.[27]

### Statistical analysis

To assess the relation between 25-hydroxyvitamin D and CRP we performed linear regression analysis. Due to its right skewed distribution, CRP levels were natural log-transformed prior to analysis. Participants with values larger than 4 standard deviations from the mean in natural log-transformed CRP (lnCRP) and/or 25-hydroxyvitamin D were excluded from the analyses.

In the first model, we assessed the association between lnCRP and 25-hydroxyvitamin D in samples taken from RSI-3, RSII-1 and RSIII-1, adjusting for age, sex and cohort. In the second model, additional adjustments were made for variables including body mass index (BMI), total cholesterol to high-density lipoprotein ratio (TC/HDL ratio), systolic blood pressure (SBP), smoking status, alcohol intake, estimated glomerular filtration rate (eGFR), prevalent type 2 diabetes mellitus (DM), season of blood drawing and level of education. We also performed stratified linear regression analysis for deficient (<50 nmol/l), insufficient (50–75 nmol/l) and sufficient (>75 nmol/l) plasma levels of vitamin D, in accordance with the guidelines of the Endocrine Society.[28] Additionally, we repeated these analyses in a quadratic model, in which we added a variable for squared 25-hydroxyvitamin D to assess whether the relation between 25-hydroxyvitamin D and CRP was non-linear. To account for potential confounding by use of vitamin D supplements, we repeated our analyses in a subset of RSI-3 (n = 2,746), which we adjusted for prevalent osteoporosis as a proxy for supplement use.

We constructed a genetic risk score (GRS) by adding the 25-hydroxyvitamin D lowering alleles (coded 0–2) from each selected SNP for each individual. [22] For C-reactive protein, we created a similar genetic risk score from 18 CRP related SNPs, with the effect allele being the CRP raising allele.[23] We performed linear regression analysis to confirm the association between the genetic risk scores and their respective phenotypes. We then performed bi-directional Mendelian randomization analyses. First, we tested the associations between individual 25-hydroxyvitamin D related SNPs and lnCRP and corrected them using Bonferroni correction.[29] We used age, sex and cohort adjusted linear regression to examine the effect of the GRS for 25-hydroxyvitamin D on lnCRP and the effect of the GRS for CRP on 25-hydroxyvitamin D. Furthermore, we used a method proposed by Dastani et al. to approximate the effect of the GRS for 25-hydroxyvitamin D on lnCRP using data of a CRP GWAS with a sample size of 66,185 so we would be able to achieve greater power.[23, 30]

For all but one variable, less than 2% of participants had missing data. For alcohol intake the percentage missing was 6.7%. We used multiple imputation, creating 5 datasets, to complete cases with missing values for the variables included in our analysis. We did not impute 25-hydroxyvitamin D or C-reactive protein levels, but we did enter them as predictor variables in our imputation model. An overview of missing data is given in S1 Table.

Tests were considered statistically significant at p-values lower than 0.05. Analyses were performed with IBM SPSS Statistics version 21.0.

## Results

Characteristics of the population under study are shown in Table 2, categorized according to vitamin D status. The mean age of the participants was 64.9 years and 43.2% were male. The mean plasma 25-hydroxyvitamin D level was 55.9 nmol/l (SD 27.6) and median CRP was 1.6 mg/l (IQR: 0.70–3.55). Study participants that had data on 25-hydroxyvitamin D available (n = 9,649) were divided in groups of sufficient vitamin D levels (n = 2,294), insufficient levels (n = 2,784) or deficient levels (n = 4,571). Participants from the population eligible for analysis were younger, had lower blood pressure, a lower prevalence of diabetes and a higher education than those from the non-eligible population (S2 Table). After correcting for age, the differences in systolic blood pressure and alcohol intake disappeared.

**Table 2:** Characteristics of study participants.

	<50 nmol/l	50–75 nmol/l	>75 nmol/l
**Number of subjects**	4,571	2,784	2,294
**Age, years**	70.9 (10.7)	63.5 (8.7)	62.1 (7.9)
**Sex, male**	1,725 (37.7)	1,303 (46.8)	1,139 (49.7)
**Body mass index, kg/m** ^**2**^	28 (5)	27 (4)	26 (4)
**25-hydroxyvitamin D, nmol/l**	32.6 (10.6)	61.8 (7.1)	95.0 (16.5)
**C-reactive protein, mg/l**	2.0 (0.8–4.1)	1.4 (0.6–3.1)	1.2 (0.5–2.7)
**Systolic blood pressure, mmHg**	141 (22)	138 (20)	136 (20)
**eGFR, ml/min/1,73m** ^**2**^	81 (19)	82 (17)	82 (16)
**TC/HDL ratio**	4.5 (1.4)	4.3 (1.3)	4.2 (1.3)
**Alcohol intake, gram/day**	5.7 (0.3–15.0)	15.0 (1.4–16.3)	15.0 (2.9–24.3)
**Smoking**			
** Never**	1,504 (32.9)	799 (28.7)	623 (27.2)
** Former**	1,931 (42.2)	1,388 (49.9)	1,156 (50.4)
** Current**	1,064 (23.3)	566 (21.0)	499 (21.8)
**Prevalent DM**	701 (15.3)	272 (9.8)	148 (6.5)
**Level of education**			
** ISCED 0**	692 (15.1)	286 (10.3)	225 (9.8)
** ISCED 1**	1,838 (40.2)	1,130 (40.6)	904 (39.4)
** ISCED 2**	1,275 (27.5)	806 (29.0)	714 (31.1)
** ISCED 3**	742 (16.2)	548 (19.7)	424 (18.5)

Numbers show mean (SD) for age, body mass index, 25-hydroxyvitamin D, systolic blood pressure, eGFR and TC/HDL ratio, median (IQR) for C-reactive protein and alcohol intake, and frequency (%) for sex, smoking, prevalent DM and level of education

Abbreviations: eGFR = estimated glomerular filtration rate; TC/HDL ratio = total cholesterol to high-density lipoprotein ratio; DM = diabetes mellitus; ISCED = International Standard Classification of Education

Table 3 shows the results of the linear regression analysis of lnCRP on 25-hydroxyvitamin D. In the age, sex and cohort adjusted linear regression, lnCRP decreased with 0.13 unit (95% CI: -0.15, -0.11) per standard deviation increase in 25-hydroxyvitamin D. There was a consistent trend across the three different categories of vitamin D levels (p = 4.98∙10^−25^). After further adjustment for BMI, SBP, eGFR, TC/HDL ratio, alcohol intake, smoking, prevalent diabetes, season of blood drawing, income and level of education, the effect estimates attenuated substantially (B = -0.06, 95% CI: -0.08, -0.03, p for trend = 4.48∙10^−6^).

**Table 3:** Association between serum 25-hydroxyvitamin D and C-reactive protein.

	N	Model 1	Model 2
		Beta (95% CI)	Beta (95% CI)
<50 nmol/l	4,571	Reference	Reference
50–75 nmol/l	2,784	-0.23 (-0.28, -0.18)	-0.12 (-0.17, -0.07)
>75 nmol/l	2,294	-0.28 (-0.34, -0.22)	-0.12 (-0.18, -0.07)
P for trend		4.98×10^−25^	4.48×10^−6^
Per SD 25OHD*	9,649	-0.13 (-0.15, -0.11)	-0.06 (-0.08, -0.03)
P-value		2.31×10^−27^	1.70×10^−6^

Model 1: adjusted for age, sex and cohort

Model 2: adjusted for age, sex, cohort, body mass index, total cholesterol to high-density lipoprotein ratio, systolic blood pressure, prevalent diabetes mellitus, estimated glomerular filtration rate, smoking, alcohol intake, season and level of education

*25OHD denotes 25-hydroxyvitamin D

We repeated these analyses with a quadratic term for vitamin D added to the regression model. Squared vitamin D was significantly associated with log-transformed CRP in both the first (p = 8.55∙10^−9^) and the second model (p = 3.21∙10^−6^) (S3 Table).

Moreover, in a subset of RSI-3 in which we additionally adjusted for osteoporosis, we found similar results in the first and second model as in the previous analyses comprising the larger study population (Table 4). Our quadratic model was not significant in this subset (S4 Table).

**Table 4:** Association between serum 25-hydroxyvitamin D and C-reactive protein in subjects with data on osteoporosis available.

	N	Model 1	Model 2	Model 3
		Beta (95% CI)	Beta (95% CI)	Beta (95% CI)
<50 nmol/l	1,579	Reference	Reference	Reference
50–75 nmol/l	749	-0.22 (-0.31, -0.12)	-0.12 (-0.21, -0.03)	-0.12 (-0.21, -0.03)
>75 nmol/l	418	-0.26 (-0.37, -0.14)	-0.15 (-0.26, -0.04)	-0.15 (-0.26, -0.04)
P for trend		6.15×10^−7^	0.003	0.003
Per SD 25OHD*	2,746	-0.12 (-0.17, -0.08)	-0.07 (-0.12, -0.03)	-0.07 (-0.11, -0.02)
P-value		5.48×10^−7^	0.004	0.004

Model 1: adjusted for age and sex

Model 2: adjusted for age, sex, body mass index, total cholesterol to high-density lipoprotein ratio, systolic blood pressure, prevalent diabetes mellitus, estimated glomerular filtration rate, smoking, alcohol intake, season and level of education

Model 3: additionally adjusted for osteoporosis

* 25OHD denotes 25-hydroxyvitamin D.

### Mendelian randomization analyses

**Fig 2:**
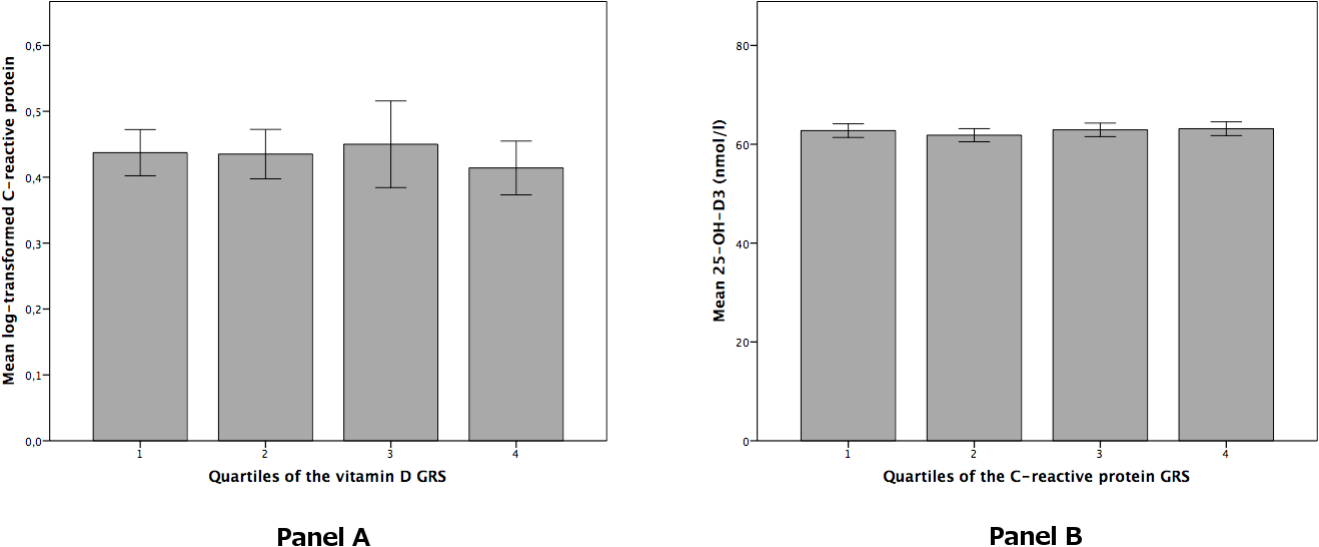
Results of Mendelian randomization analyses with the genetic risk scores in quartiles. Panel A: quartiles of the 25-hydroxyvitamin D genetic risk score in relation to C-reactive protein. P for trend = 0.056. Panel B: quartiles of the C-reactive protein genetic risk score in relation to 25-hydroxyvitamin D. P for trend = 0.374Error bars represent 95% confidence intervals.

The genetic risk scores for vitamin D and CRP were robustly associated with their respective phenotypes (S1 and S2 Figs). The 25-hydroxyvitamin D GRS explained 5.1% of the variation in serum 25-hydroxyvitamin D. The 25-hydroxyvitamin D GRS was not associated with lnCRP (n = 10,788, β = -0.018 per SD, p = 0.082). Moreover, there was no significant trend across the GRS quartiles (Fig 2). Associations of individual SNPs with lnCRP are shown in S5 Table. Among all, rs2282679 (*GC*: Vitamin D binding protein) was significantly associated with lnCRP (p = 0.027), however, after correcting for multiple testing this was no longer significant. The additional analysis that estimated the effect of the GRS for 25-hydroxyvitamin D on lnCRP in data of a CRP GWAS did not provide a significant result (p = 0.23). The CRP GRS explained 5.5% of the variation in lnCRP. We did not observe a significant association between the CRP GRS and serum 25-hydroxyvitamin D (n = 6,267, β = 0.001 per SD, p = 0.998). Similarly, after dividing the GRS in quartiles, there was no significant trend (Fig 2).

## Discussion

Our observational data suggest an inverse association between serum 25-hydroxyvitamin D and C-reactive protein. However, since genetic determinants of serum vitamin D were not associated with serum CRP in the Mendelian randomization approach, our study does not provide evidence for a causal relationship between vitamin D and inflammation.

There are several ways in which vitamin D is able to affect the immune system that could explain the observed association with CRP. It has been shown that immune cells, such as macrophages and dendritic cells, express 1-a-hydroxylase, and thus are able to locally convert 25-hydroxyvitamin D into the active form of vitamin D, 1.25-dihydroxyvitamin D. [31, 32] Moreover, the vitamin D receptor is present on leukocytes, T-helper cells and monocytes. 1.25-dihydroxyvitamin D has been shown to inhibit production of inflammatory markers such as IFN-γ, IL-2, and IL-5 by T-helper 1 lymphocytes.[33, 34] Vitamin D also inhibits synthesis of IL-6 by monocytes, which is the primary stimulant of CRP production in the liver.[35, 36]

Previous observational studies that investigated the relationship between vitamin D and inflammatory markers such as CRP have shown mixed results. Shea et al. studied the relation of vitamin D with several inflammatory markers cross-sectionally in 1,381 subjects from the Framingham Offspring Study cohort and did not find a significant association for most of the markers, including CRP.[6] Another, smaller study by Michos et al. did also not find a significant association between vitamin D and CRP. [7] Patel et al. observed an inverse relation between vitamin D and CRP in patients with polyarthritis.[8] Amer et al. found a significant inverse association between 25-hydroxyvitamin D and CRP in a cross-sectional setting in a population of 15,167 adults with a mean age of 46 years from the United States. However, for vitamin D levels above the population median of 21 ng/ml, this relationship reversed, leading the authors to conclude that above this level, vitamin D may actually be pro-inflammatory. [9] In our study, we found that a quadratic model fit the data better than a linear model, suggesting that the relation between vitamin D and CRP may indeed not be linear. The analyses by Amer et al. were done in a younger population and were not adjusted for season of blood drawing or geographical location, which may explain the difference compared to our results.

Several randomized controlled trials have been performed to investigate the effect of vitamin D supplementation on CRP. Coussens et al. found that 95 patients who were treated for tuberculosis and received additional vitamin D supplementation had a faster drop in CRP levels than those who received placebo.[10] In a small study of 54 subjects by Timms et al. there was a decrease in CRP after one year of vitamin D supplementation, but the study was unblinded and included severely vitamin D deficient subjects (25-hydroxyvitamin D <11 ng/ml or <27 nmol/l) only.[11] Chen et al. performed a meta-analysis of randomized controlled trials that investigated the effect of vitamin D on high-sensitive C-reactive protein. They analyzed data of 10 studies, totaling 924 subjects, and found that vitamin D had a significant effect on C-reactive protein. Since there was evidence of heterogeneity these results should be interpreted with caution.[12] However, other randomized trials have not been able to confirm these effects. Schleithoff et al. investigated cytokine profiles in 93 heart failure patients who received vitamin D supplementation or placebo. After 9 months of follow-up there was no effect on CRP.[13] In a study of 314 subjects, Pittas et al. found that after 3 years of vitamin D supplementation there was no significant difference in the decrease of CRP between the placebo and treatment group.[14] Bjorkman et al. did not find an effect of vitamin D supplementation versus placebo in a 6-month trial in 218 older patients.[15]

High vitamin D levels may be the result of oral supplementation. Subjects that have an indication to use vitamin D supplements are generally people with decreased bone mineral density.[28] These subjects are more likely to have comorbidities, and thus increased CRP levels. Therefore, use of supplements is a possible confounder of the association between vitamin D and CRP. Since no reliable data were available for vitamin D supplementation, we used prevalent osteoporosis as a proxy for use of vitamin D supplements and adjusted for this in a sensitivity analysis. This did not influence our effect estimate. The quadratic model was not significant in this subset, possibly due to a small sample size and limited power.

Mendelian randomization analyses did not provide significant results. The association between the vitamin D GRS and lnCRP is not consistent with the observational association that we found between serum vitamin D and lnCRP, since the direction of effect is opposite. The result was mainly driven by one SNP, rs2282679, which is located in the gene that encodes the vitamin D binding protein that has no other known functions.

The major strengths of this study are the large sample size for measurements of both CRP and vitamin D, and a comprehensive assessment of this association using both observational and genetic data. By using analytic methods proposed by Dastani et al., we were able to greatly increase the number of subjects for Mendelian randomization analysis. We are the first study to investigate the causal relationship between vitamin D and inflammation through the Mendelian randomization approach. Some limitations should be acknowledged. The 25-hydroxyvitamin D GRS explained only 5.1% of the variation in serum 25-hydroxyvitamin D and the CRP GRS only explained 5.5 of the variation in serum CRP, which could mean that our study is underpowered to find a significant association in Mendelian randomization analyses. We only studied one inflammatory marker to assess the association between vitamin D and inflammation. However, CRP is a widely used marker for chronic inflammation that comprises different aspects of the complex immune system. We aimed to adjust for vitamin D supplement intake, but we did not have a representative variable and had to use a proxy on which information was only available for a small number of people. Our population consisted of elderly individuals, who have more co-morbidities than younger people and are more likely to be sun deprived, which could have had impact on our results. Furthermore, the results may not be valid for all ethnic groups, since our population consisted of Caucasian individuals.

## Conclusion

In conclusion, serum vitamin D was inversely associated with CRP, but results of Mendelian randomization analyses do not provide evidence for a causal association. The observed association between vitamin D and CRP is possibly due to residual confounding, but a causal relationship cannot be ruled out yet. Further studies are necessary to understand the role and mechanisms of vitamin D on non-communicable disease prevention and the potential effect of vitamin D supplementation on inflammation.

## Supporting Information

S1 Fig Quartiles of the 25-hydroxyvitamin D genetic risk score in relation to 25-hydroxyvitamin D (PDF)

S2 Fig Quartiles of the C-reactive protein genetic risk score in relation to C-reactive protein (PDF)

S1 Table Overview of missing data (PDF)

S2 Table Comparison of the population under study with the population not under study (PDF)

S3 Table P-values for the association between serum 25-hydroxyvitamin D and C-reactive protein in a quadratic model (PDF)

S4 Table P-values for the association between serum 25-hydroxyvitamin D and C-reactive protein in a quadratic model in subjects with data on osteoporosis available (PDF)

S5 Table Individual associations of vitamin D related SNPs with C-reactive protein (PDF)
