# Supplementary material for: Vitamin D and C-Reactive Protein: A Mendelian Randomization Study
Source: PLoS One. 2015 Jul 6;10(7):e0131740. doi: 10.1371/journal.pone.0131740 (PMC4492676; doi:10.1371/journal.pone.0131740)
Supplement: S1 Fig — (PDF) [file pone.0131740.s001.pdf]

S1 Figure. Quartiles of the 25-hydroxyvitamin D score in relation to serum 25-hydroxyvitamin D

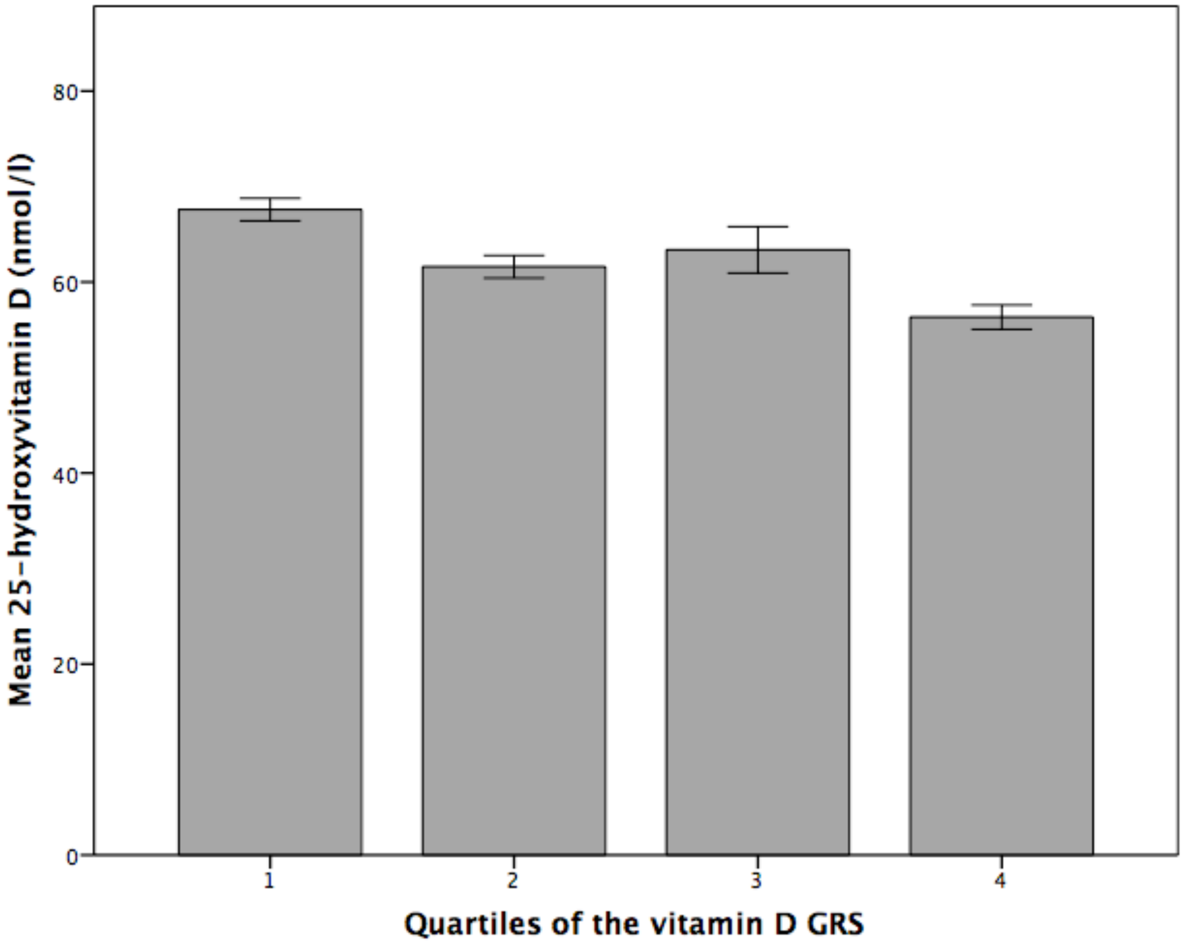

Error bars represent 95% confidence intervals

P for trend = $1.07*10^{-35}$
