# Supplementary material for: Vitamin D and C-Reactive Protein: A Mendelian Randomization Study
Source: PLoS One. 2015 Jul 6;10(7):e0131740. doi: 10.1371/journal.pone.0131740 (PMC4492676; doi:10.1371/journal.pone.0131740)
Supplement: S2 Fig — (PDF) [file pone.0131740.s002.pdf]

S2 Figure. Quartiles of the C-reactive protein GRS in relation to C-reactive protein

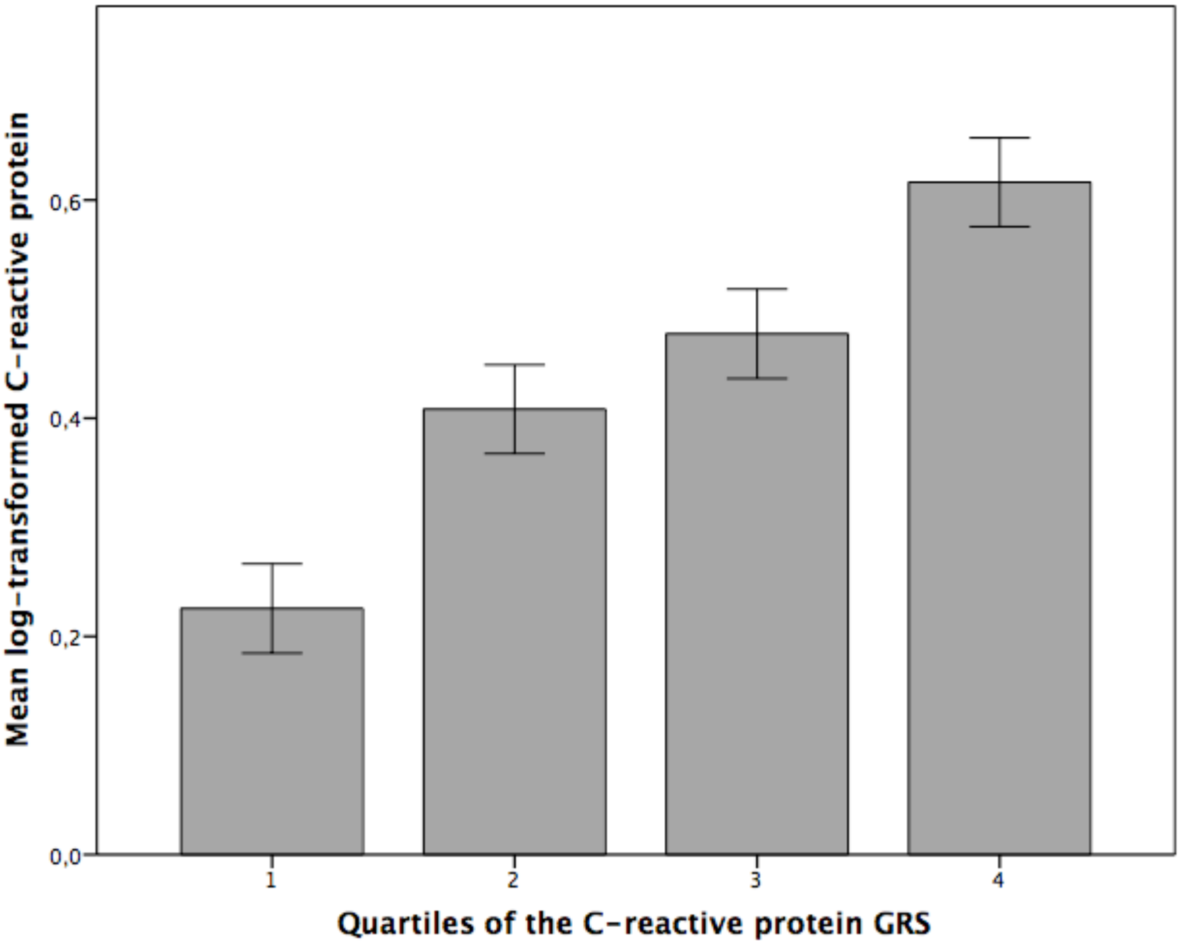

Error bars represent 95% confidence intervals

P for trend = $7.99*10^{-40}$
