# Supplementary material for: Vitamin D and C-Reactive Protein: A Mendelian Randomization Study
Source: PLoS One. 2015 Jul 6;10(7):e0131740. doi: 10.1371/journal.pone.0131740 (PMC4492676; doi:10.1371/journal.pone.0131740)
Supplement: S1 Table — (PDF) [file pone.0131740.s003.pdf]

S1 Table. Overview of missing data

Variable	Percentage missing	Imputed yes/no
Cohort	0	No
Age	0	No
Sex	0	No
25-hydroxyvitamin D	0	No
lnCRP	0	No
Body Mass Index	1.4	Yes
Systolic blood pressure	0.6	Yes
TC/HDL	1.4	Yes
Diabetes Mellitus	0.4	Yes
eGFR	1.6	Yes
Season	0.3	Yes
Alcohol intake	6.7	Yes
Smoking	1.0	Yes
Level of education	0.9	Yes

Abbreviations: lnCRP = natural log-transformed C-reactive protein; TC/HDL ratio = total cholesterol/high density lipoprotein ratio; eGFR = estimated glomerular filtration rate
