# Supplementary material for: Vitamin D and C-Reactive Protein: A Mendelian Randomization Study
Source: PLoS One. 2015 Jul 6;10(7):e0131740. doi: 10.1371/journal.pone.0131740 (PMC4492676; doi:10.1371/journal.pone.0131740)
Supplement: S2 Table — (PDF) [file pone.0131740.s004.pdf]

S2 Table. Comparison of the population under study with the population not under study

	Population for analysis	Population not eligible for analysis	p-value
**Number**	9,649	4,977	
**Age, years**	64.9 (9.8)	73.5 (10.8)	<0.001
**Sex, male**	4,167 (43.2)	1,860 (37.4)	<0.001
**Body mass index, kg/m^2**	27 (5)	27 (4)	0.927
**Systolic blood pressure, mmHg**	140 (21)	142 (23)	0.001
**eGFR, ml/min/1,73m^2**	81.2 (17.9)	80.4 (18.6)	0.392
**TC/HDL ratio**	4.4 (1.4)	4.4 (1.3)	0.841
**Alcohol Intake, gram/day**	12.1 (0.7-15.0)	2.9 (0.0-15.0)	<0.001
**Smoking**			<0.001
**Never**	2,926 (30.3)	641 (12.9)	
**Former**	4,475 (46.4)	796 (16.0)	
**Current**	2,129 (22.5)	484 (9.7)	
**Prevalent DM**	1,121 (11.6)	695 (14.0)	<0.001
**Level of education**			<0.001
**ISCED 0**	1,203 (12.5)	1,458 (29.9)	
**ISCED 1**	3,872 (40.1)	1,841 (37.0)	
**ISCED 2**	2,777 (28.8)	1,064 (21.4)	
**ISCED 3**	1,714 (17.8)	402 (8.1)	

Numbers show mean (SD) for age, body mass index, systolic blood pressure, eGFR and TC/HDL ratio, median (IQR) for alcohol intake, and frequency (%) for sex, smoking, prevalent DM and level of education

Abbreviations: eGFR = estimated glomerular filtration rate; TC/HDL ratio = total cholesterol to high-density lipoprotein ratio; DM = diabetes mellitus; ISCED = International Standard Classification of Education
