# Supplementary material for: Vitamin D and C-Reactive Protein: A Mendelian Randomization Study
Source: PLoS One. 2015 Jul 6;10(7):e0131740. doi: 10.1371/journal.pone.0131740 (PMC4492676; doi:10.1371/journal.pone.0131740)
Supplement: S3 Table — (PDF) [file pone.0131740.s005.pdf]

S3 Table. P-values for the association between serum 25-hydroxyvitamin D and C-reactive protein in a quadratic model

	N	Model 1	Model 2
**Squared 25OHD***	9,649	$p = 8.55\times10^{-9}$	$p = 3.21\times10^{-6}$

Model 1: adjusted for age, sex and cohort

Model 2: adjusted for age, sex, cohort, body mass index, total cholesterol to high-density lipoprotein ratio, systolic blood pressure, prevalent diabetes mellitus, estimated glomerular filtration rate, smoking, alcohol intake, season and level of education

*25OHD denotes 25-hydroxyvitamin D
