# Supplementary material for: Vitamin D and C-Reactive Protein: A Mendelian Randomization Study
Source: PLoS One. 2015 Jul 6;10(7):e0131740. doi: 10.1371/journal.pone.0131740 (PMC4492676; doi:10.1371/journal.pone.0131740)
Supplement: S4 Table — (PDF) [file pone.0131740.s006.pdf]

**S4 Table. P-values for the association between serum 25-hydroxyvitamin D and C-reactive protein in a quadratic model in subjects with data on osteoporosis available**

| | N | Model 1 | Model 2 | Model 3 |
|---|---|---|---|---|
| **Squared 25OHD*** | 2,746 | p = 0.153 | p = 0.333 | p = 0.336 |

Model 1: adjusted for age and sex

Model 2: adjusted for age, sex, body mass index, total cholesterol to high-density lipoprotein ratio, systolic blood pressure, prevalent diabetes mellitus, estimated glomerular filtration rate, smoking, alcohol intake, season and level of education

Model 3: additionally adjusted for osteoporosis

*25OHD denotes 25-hydroxyvitamin D
