# Supplementary material for: Vitamin D and C-Reactive Protein: A Mendelian Randomization Study
Source: PLoS One. 2015 Jul 6;10(7):e0131740. doi: 10.1371/journal.pone.0131740 (PMC4492676; doi:10.1371/journal.pone.0131740)
Supplement: S5 Table — (PDF) [file pone.0131740.s007.pdf]

S5 Table. Individual associations of vitamin D related SNPs with lnCRP

SNP	Beta	p-value
rs12785878	-0.003	0.897
rs10741657	-0.007	0.659
rs2282679	-0.036	0.027
rs6013897	-0.012	0.493

After Bonferroni correction the threshold for significance lies at p = 0.0125
